# Supplementary material for: Mapping and identification of soft corona proteins at nanoparticles and their impact on cellular association
Source: Nat Commun. 2020 Sep 10;11:4535. doi: 10.1038/s41467-020-18237-7 (PMC7484794; doi:10.1038/s41467-020-18237-7)
Supplement: Supplementary file 1 — Supplementary information [file 41467_2020_18237_MOESM1_ESM.pdf]

Supplementary Information

Mapping and Identification of soft corona proteins at nanoparticles and their impact on cellular association

Mohammad-Beigi et al

Supplementary Figure 1.

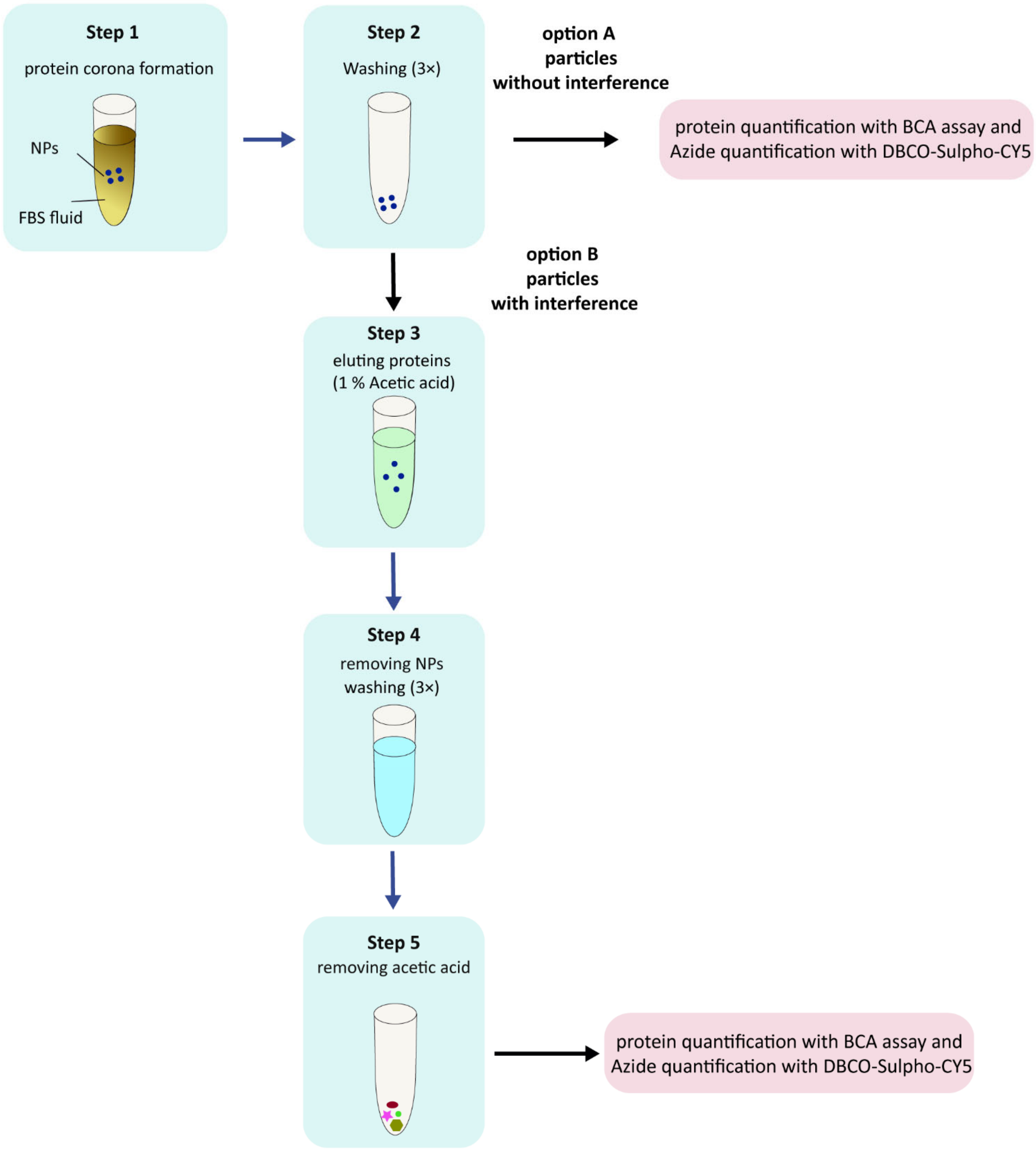

Supplementary Figure 1. Schematic overview of the protocols developed for quantification of proteins and azide groups on both particles with and without interference. For nanoparticles that interfere with the BCA assay and azide quantification with DBCO-Sulpho-CY5, we developed a method to first elute the proteins from nanoparticles and then analyse with BCA and azide quantification assays. We developed this method for SNPs, but it can be applied to all other types of nanoparticles. The results of the BCA assay and the click reaction showed the same efficiency for both eluting and non-eluting protocols.

Supplementary Figure 2.

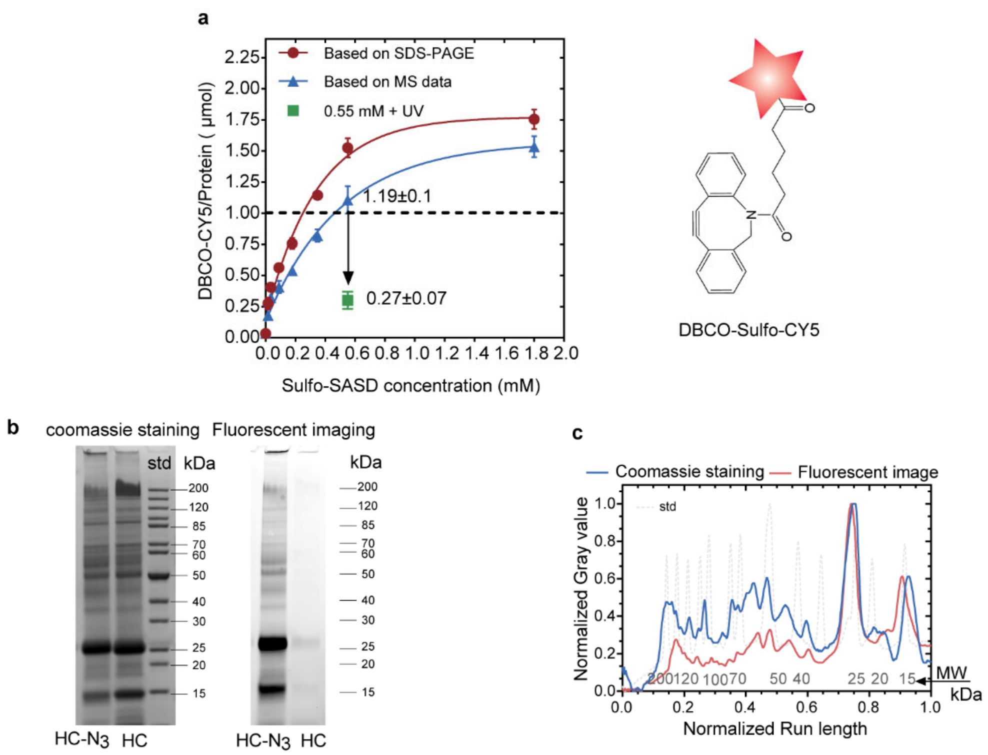

Supplementary Figure 2. Optimization of Azide modification of HC proteins on SNPs with Sulpho-SASD. a, SNPs-hard corona complexes (0.4 mg ml^{-1}) were incubated with different concentrations of Sulpho-SASD (0, 0.018, 0.036, 0.09, 0.18, 0.35, 0.55, 1.8 mM) for 1 h. Sulpho-SASD modifies protein through the reaction of its Sulpho-NHS with primary amines on proteins. To confirm the labelling, the azide-modified particles were incubated with DBCO-Sulpho-CY5 which reacts with the azide groups through a SPAAC click reaction. The number of proteins for calculation of labelling efficiency was measured using two methods. In the first method, the amount of protein was calculated by a combination of SDS-PAGE and BCA assay. In the second method, the LC-MS/MS data was used to calculate the protein content on SNPs. The results show that the labelling efficiency increases with Sulpho-SASD concentration and reaches a plateau at 0.55 mM Sulpho-SASD. Converting N_3 group by UV to nitren group decreased the click reaction efficiency, which is considered as another control experiment. Sulpho-SASD at 0.6 mM was used for further steps. Quantification data represented as the mean ± sd. of three independent experiments (n=3). **b,c,** N_3 modification of HC proteins formed on SNPs over 2 h incubation with FBS was characterized by a click reaction between N_3 and DBCO-Sulpho-CY5. Fluorescence image of the SDS-PAGE (**b**) and the comparison between the densitometry of coomassie staining and fluorescence image (**c**) show that all the proteins stained with coomassie reacted with DBCO, which confirms the presence of N_3 on all HC proteins. No CY5 fluorescence was detected for the un-labelled corona proteins in the fluorescence image, which indicates that DBCO-CY5 only reacts with N_3 modified proteins. Similar results were obtained in 3 independent experiments. Source data are provided as a Source Data file.

Supplementary Figure 3.

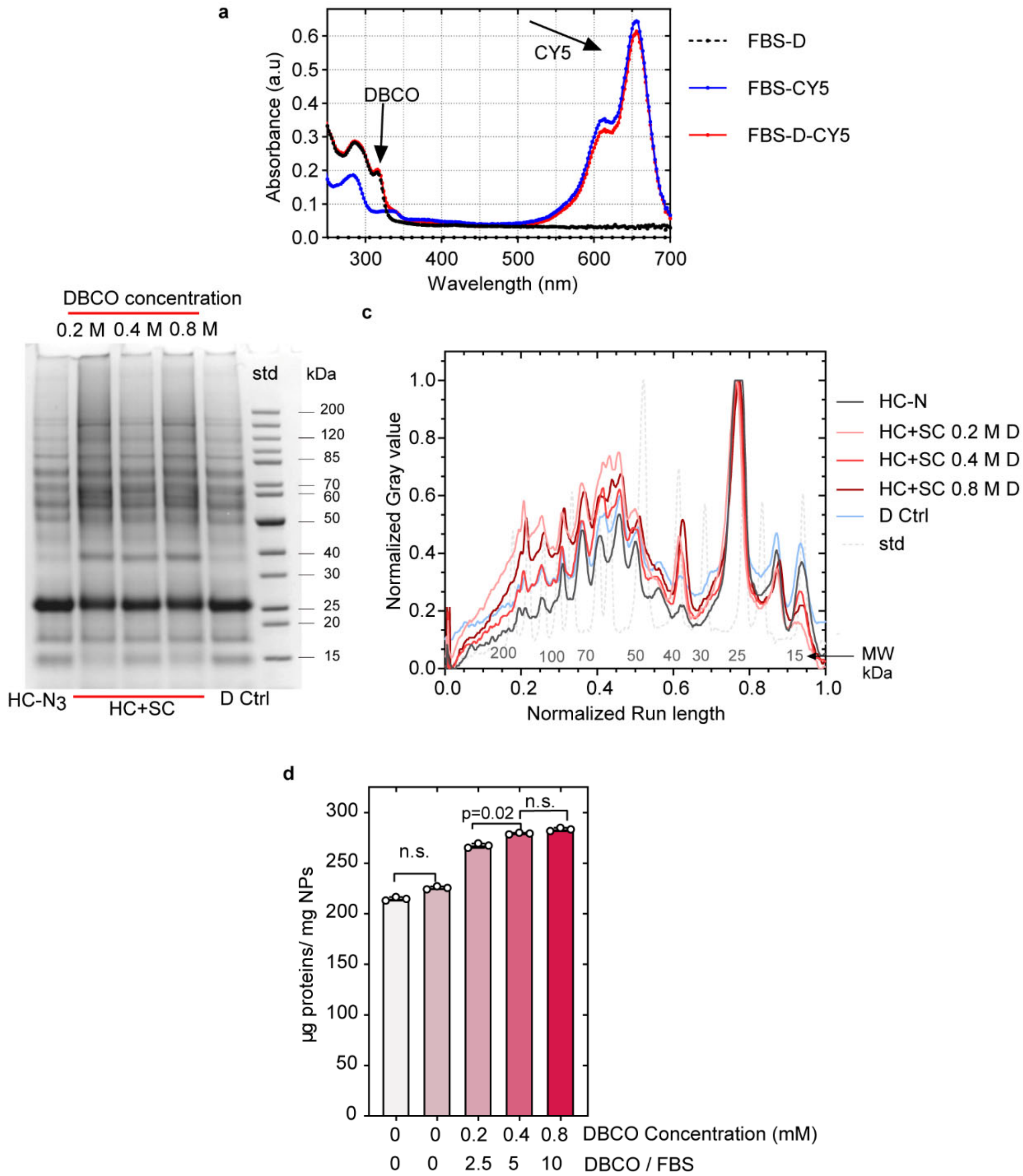

Supplementary Figure 3. Optimization of DBCO modification of FBS proteins with DBCO-Sulpho-NHS. a, UV-vis spectroscopy analysis of FBS proteins labelled with DBCO or CY5. The degree of labelling (DOL) in the Supplementary Table.S1 show that labelling efficiency does not change when the proteins were labelled with both DBCO and CY5. CY5 was only used for fluorescent detection of capture proteins in Supplementary Figure 4. **b-d,** Different DBCO concentrations (0, 0.2, 0.4, and 0.8 mM equals to 0, 2.5, 5, and 10 of DBCO/FBS) were used for modification of FBS proteins. Then, the FBS-D proteins were added to the N_3 modified HC proteins on SNPs. The corona protein was visualized by a SDS-PAGE gel (**b**), analysed with densitometry analysis (**c**), and quantified by BCA assay (**d**). The SDS-PAGE analysis shows addition of some proteins by the click reactions. Similar results were obtained in 3 independent experiments. The DOL increases from 4.2±0.3 to 5.1±0.2 by increasing DBCO-Sulpho-NHS concentration from 0.2 to 0.4, which only increases to 5.6±0.1 when the concentration is 0.8 mM. Quantification data represented as the mean ± sd. of three independent experiments (n=3). For the multiple comparison, P value was calculated by one- way ANOVA with Tukey Post hoc test without any adjustment. n.s., not significant (p>0.05). Source data are provided as a Source Data file.

Supplementary Figure 4.

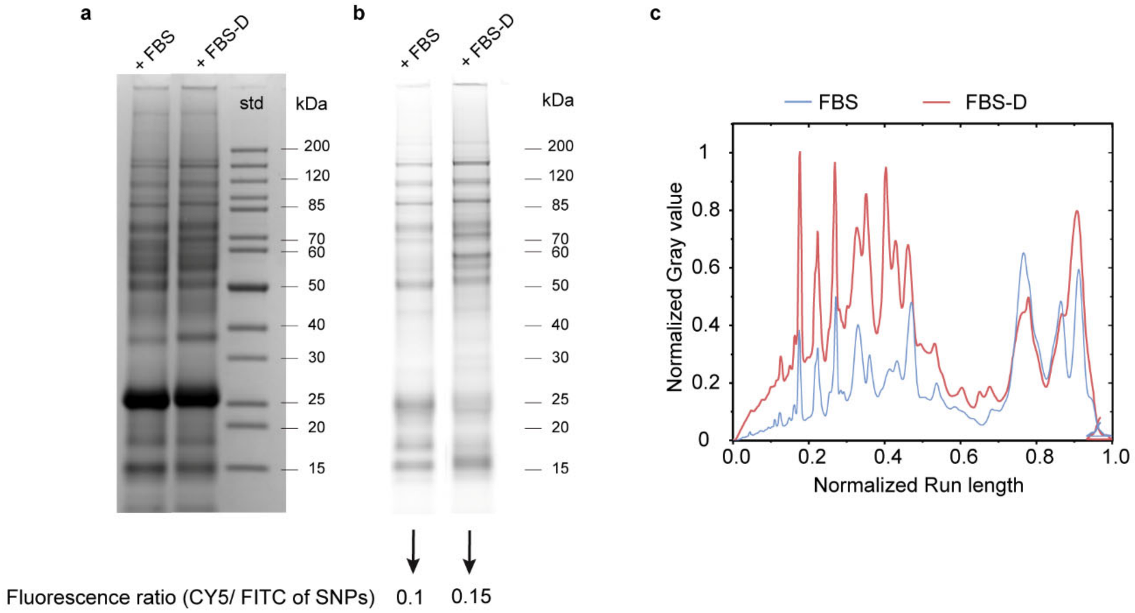

Supplementary Figure 4. Addition of fluorescently labelled FBS to SNPs@HC-N_3. a-c, Coomassie staining (**a**), fluorescence image (**b**), and densitometry analysis of the fluorescence image of SDS-PAGE gel of fluorescently labelled FBS (FBS-CY5) and FBS-D (FBS-D-CY5) proteins added to SNPS@HC-N_3. Densitometry analysis clearly shows the addition of more proteins through click chemistry to HC on SNPs. The fluorescence ratio of CY5/FITC of SNPs was also measured in both conditions (showed below the SDS-PAGE image), which confirms the addition of more fluorescently labelled proteins to HC through click chemistry. The SDS-PAGE analysis was repeated 3 times independently with similar results. Source data are provided as a Source Data file.

Supplementary Figure 5.

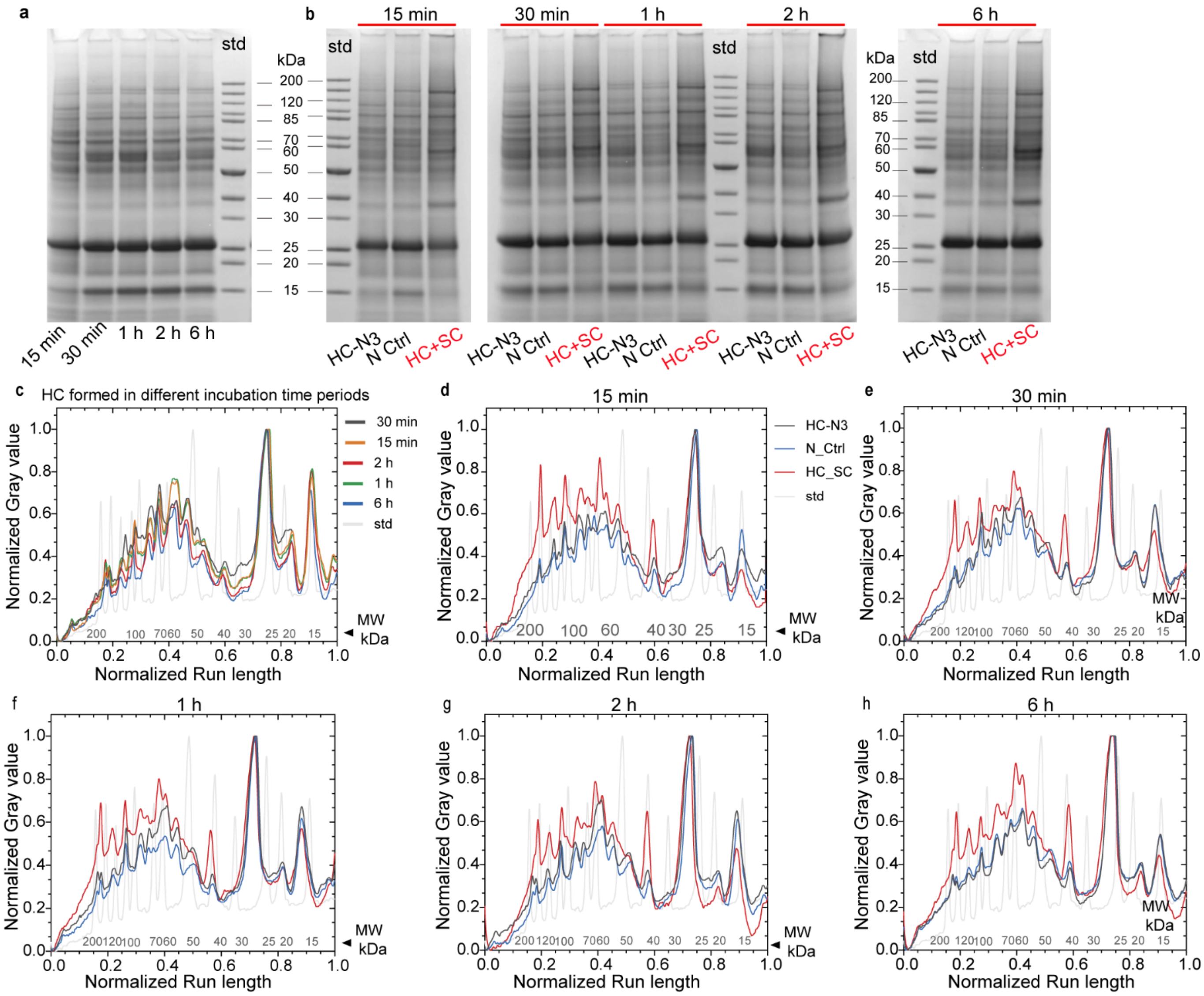

Supplementary Figure 5. Capturing weakly interacting proteins on HC proteins, which were formed by exposure to FBS for the indicated time periods (15 min, 30 min, 1h, 2h, and 6 h). a,b, SDS-PAGE of HC formed on SNPs over different exposure time periods (**a**) and HC+SC proteins on SNPs **(b).** The SDS-PAGE analysis was done two times independently with similar results. **c**, Densitometry analysis of SDS-PAGE gel in (**a**). **d-h,** Densitometry analysis of SDS-PAGE gels shown in (**b**). Source data are provided as a Source Data file.

Supplementary Figure 6.

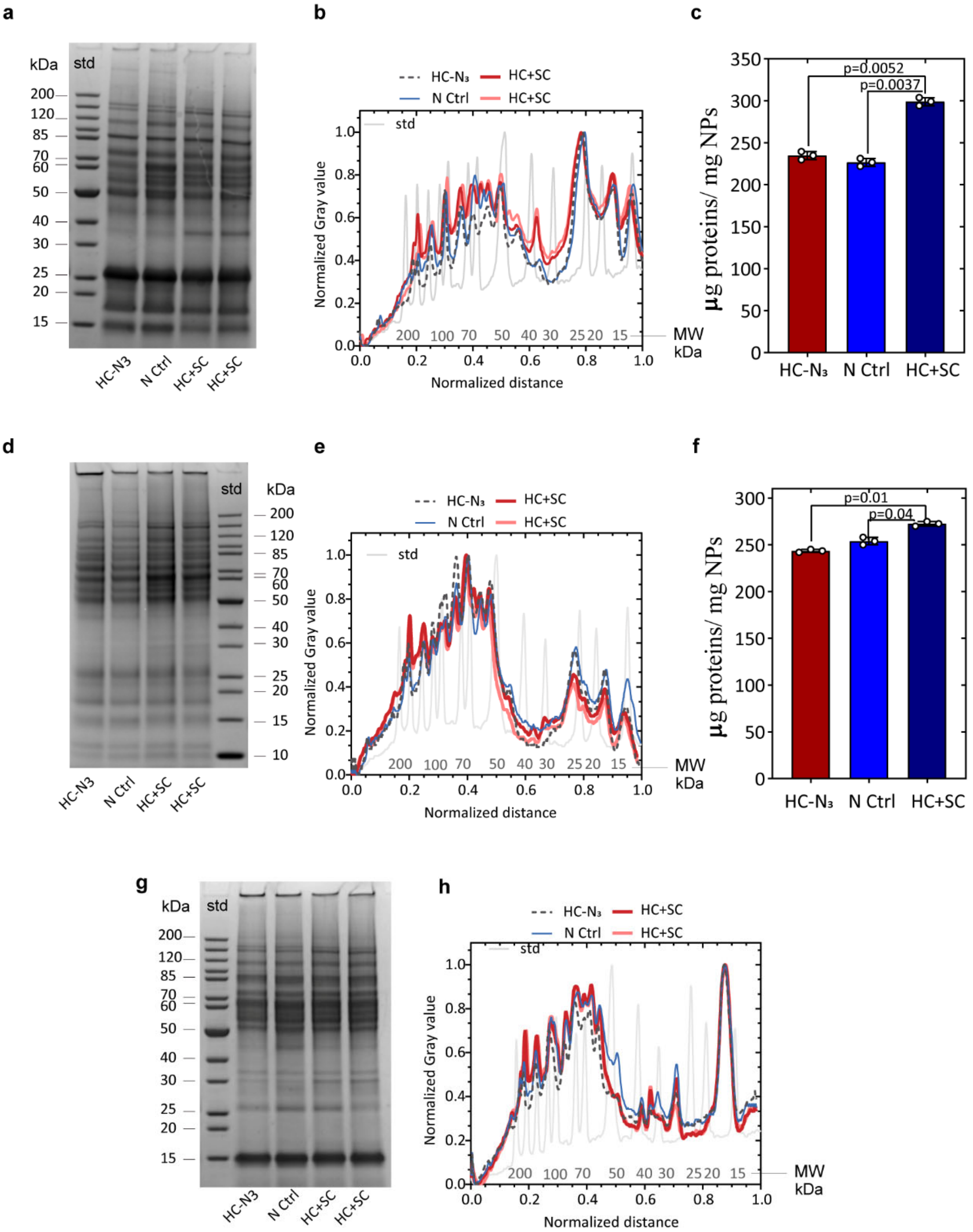

Supplementary Figure 6. Analysis of eluted corona proteins from NPs. SDS-PAGE image, densitometry analysis, and quantification of eluted corona proteins (HC and HC+SC) from amine-modified SNPs (SANPs) (**a-c**), carboxyl-modified SNPs SCNPs (**d-f**) and PsNPs (**g,h**) which were captured through click chemistry. For the all nanoparticles, SDS-PAGE analysis was repeated 3 times independently with similar results. Quantification data in **c** and **f** represented as the mean ± sd. of three independent experiments (n=3). For the multiple comparison, P value was calculated by one- way ANOVA with Tukey Post hoc test without any adjustment. Source data are provided as a Source Data file.

Supplementary Figure 7.

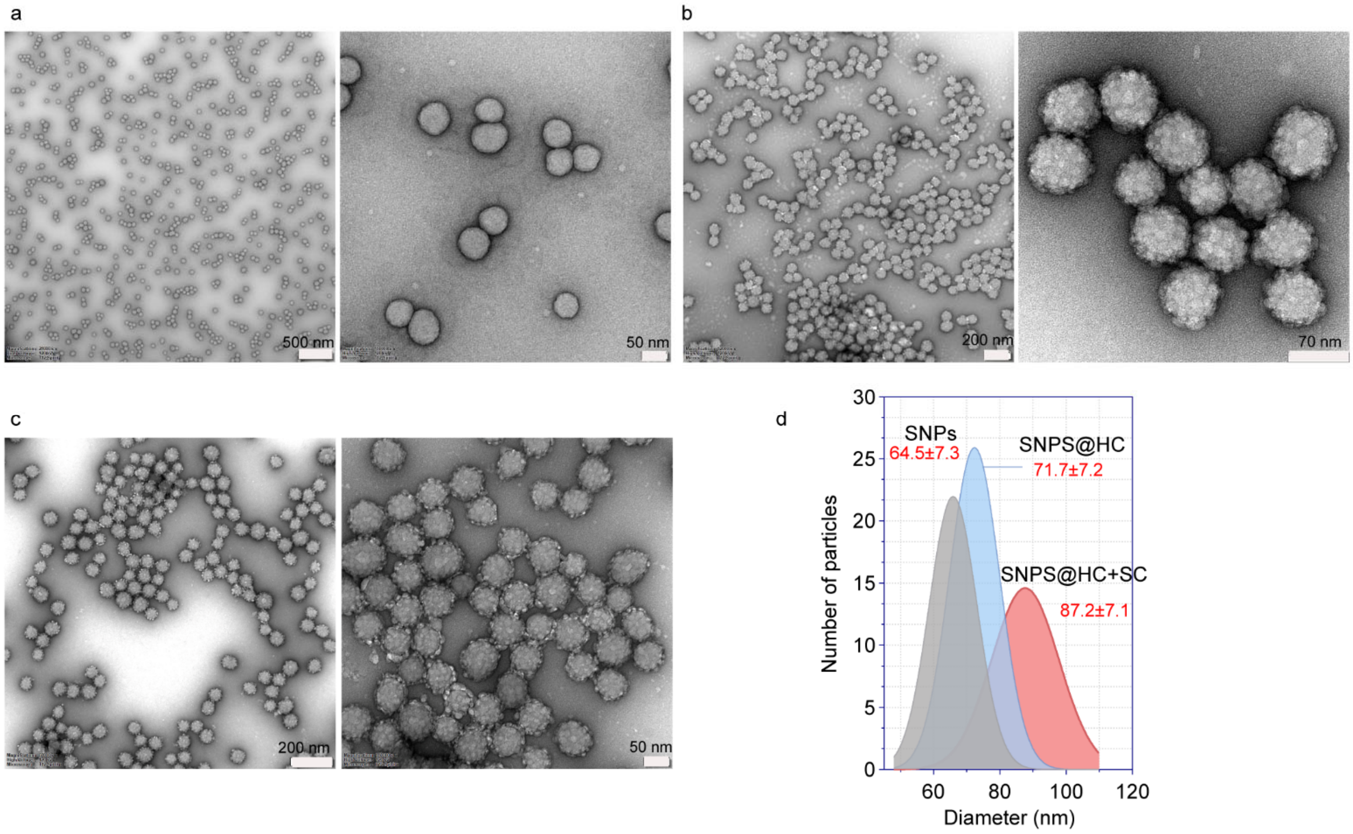

Supplementary Figure 7. Characterization of nanoparticle-corona complexes. TEM analysis of pristine SNPs (**a**), SNPs@HC (**b**), and SNPs@HC+SC (**c**). **d**, The average size of nanoparticles was calculated by measuring the size of at least 150 particles and data shown correspond to mean ± sd. TEM analysis was performed 3 times independently with similar results. Source data are provided as a Source Data file.

Supplementary Figure 8.

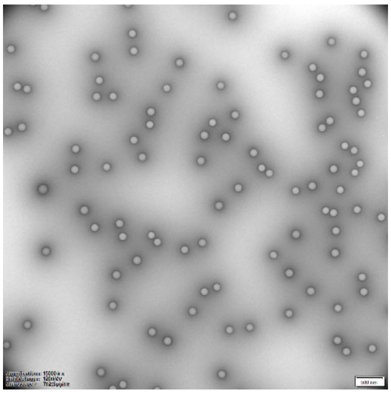

Supplementary Figure 8. TEM analysis of pristine PsNPs. Scale bar, 500 nm. TEM analysis was performed 3 times independently with similar results.

Supplementary Figure 9.

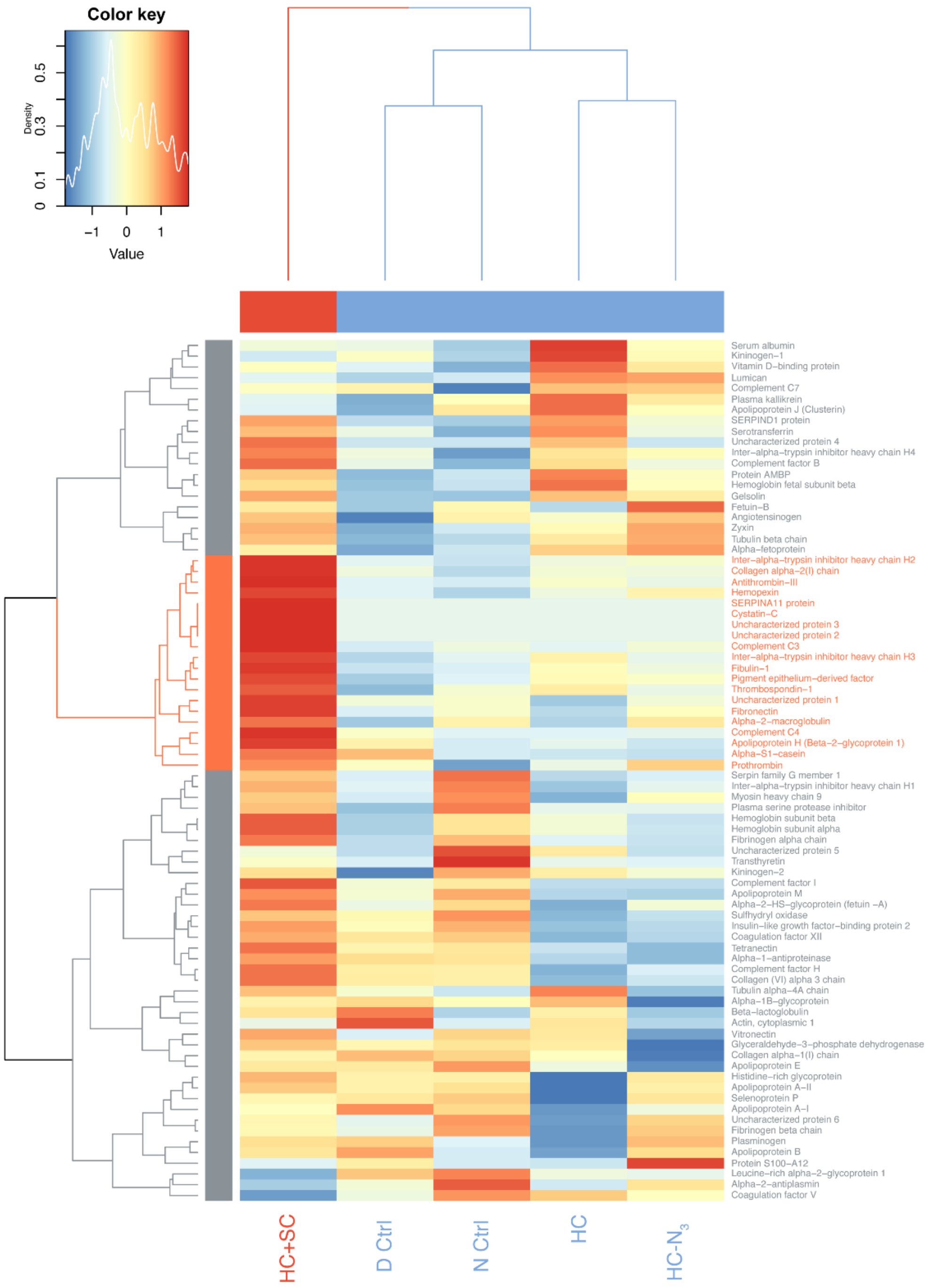

Supplementary Figure 9. Identification of corona proteins on SNPs .A heatmap with two-way unsupervised hierarchical clustering analysis (UHCA) of the relative abundance of corona proteins recovered from SNPs. Each row, a protein; each column, a protein corona sample. The number of proteins per nanoparticle is scaled to derive a z-score representing the relative abundance of each protein between the samples. A colour key along with the z-score distribution is depicted to the top left. Red and blue correspond to the number of proteins higher and lower than the average across all samples, respectively. The row dendrogram reveals a putative SC cluster (coloured in orange) characterized by specific enrichment of the proteins in HC+SC. Source data are provided as a Source Data file.

Supplementary Figure 10.

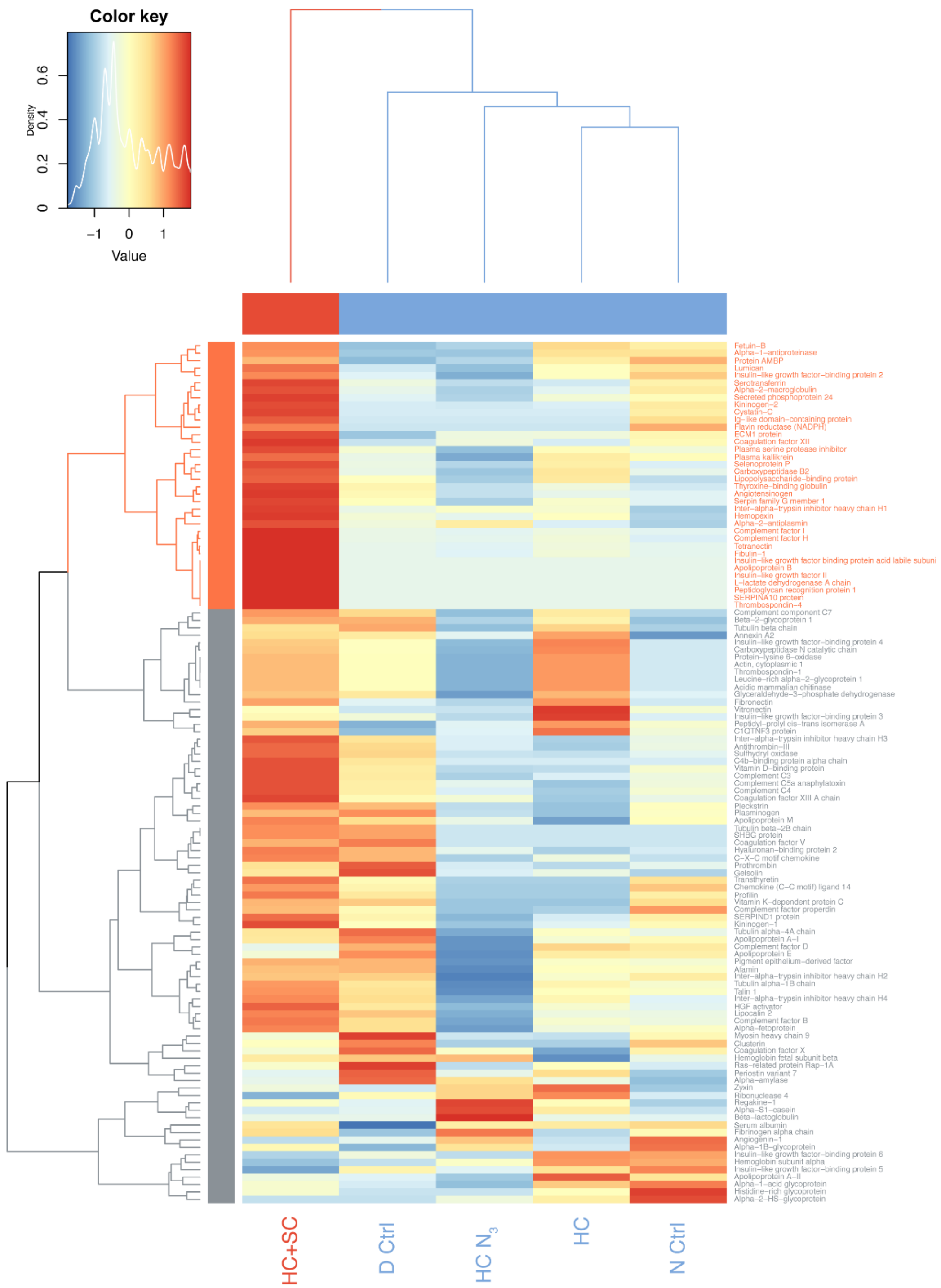

Supplementary Figure 10. Identification of corona proteins on PsNPs. A heatmap with two-way unsupervised hierarchical clustering analysis (UHCA) of the relative abundance of corona proteins recovered from PsNPs. Each row, a protein; each column, a protein corona sample. The number of proteins per nanoparticle is scaled to derive a z-score representing the relative abundance of each protein between the samples. A colour key along with the z-score distribution is depicted to the top left. Red and blue correspond to the number of proteins higher and lower than the average across all samples, respectively. The row dendrogram reveals a putative SC cluster (coloured in orange) characterized by specific enrichment of the proteins in HC+SC. Source data are provided as a Source Data file.

Supplementary Figure 11.

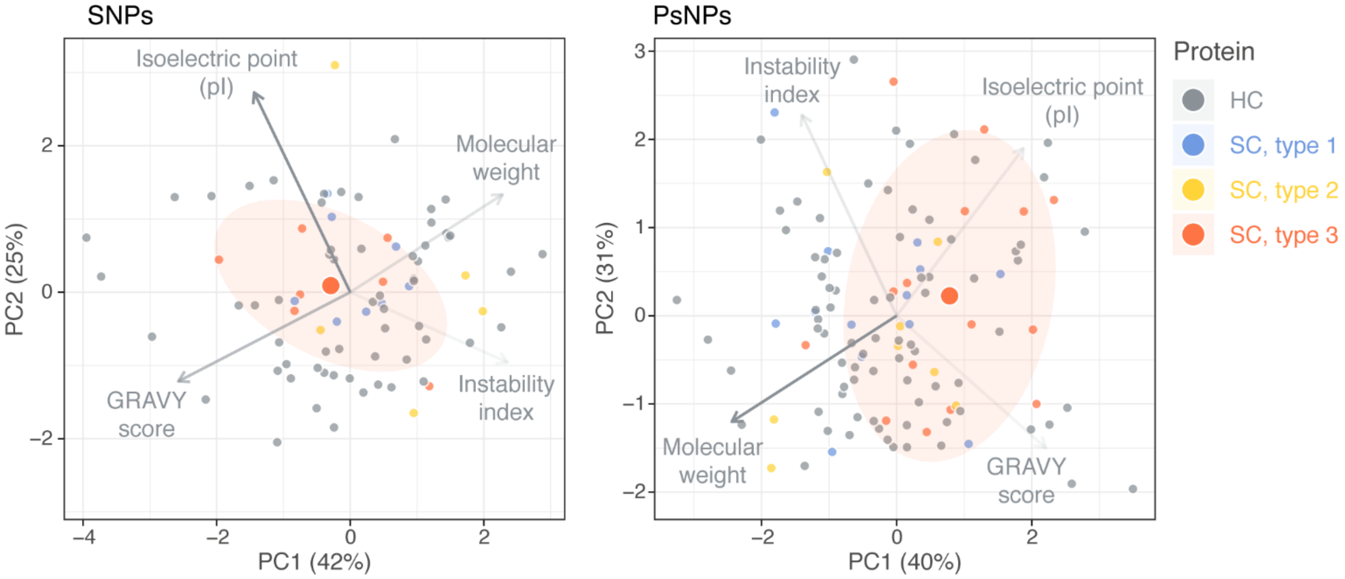

Supplementary Figure 11. Parameter analysis of corona proteins eluted from SNPs and PsNPs. Source data are provided as a Source Data file.

Supplementary Figure 12.

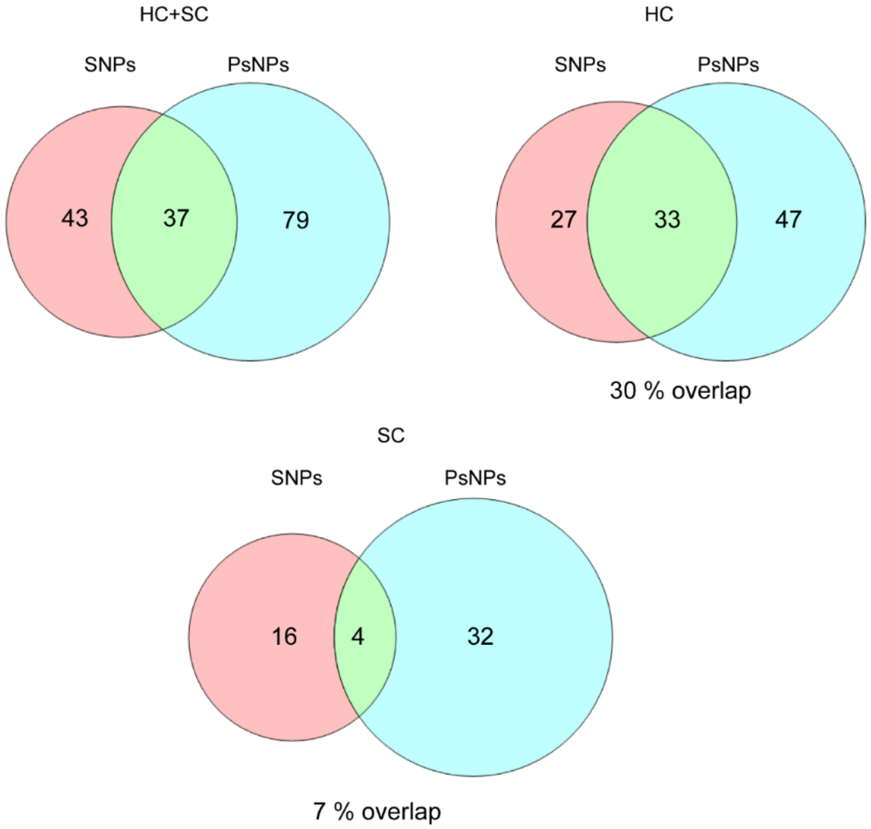

Supplementary Figure 12. Venn diagrams depicting the degree of overlap of corona proteins on SNPs and PsNPs. HC+SC (total corona proteins), HC (hard corona proteins), and SC (soft corona proteins).

Supplementary Figure 13.

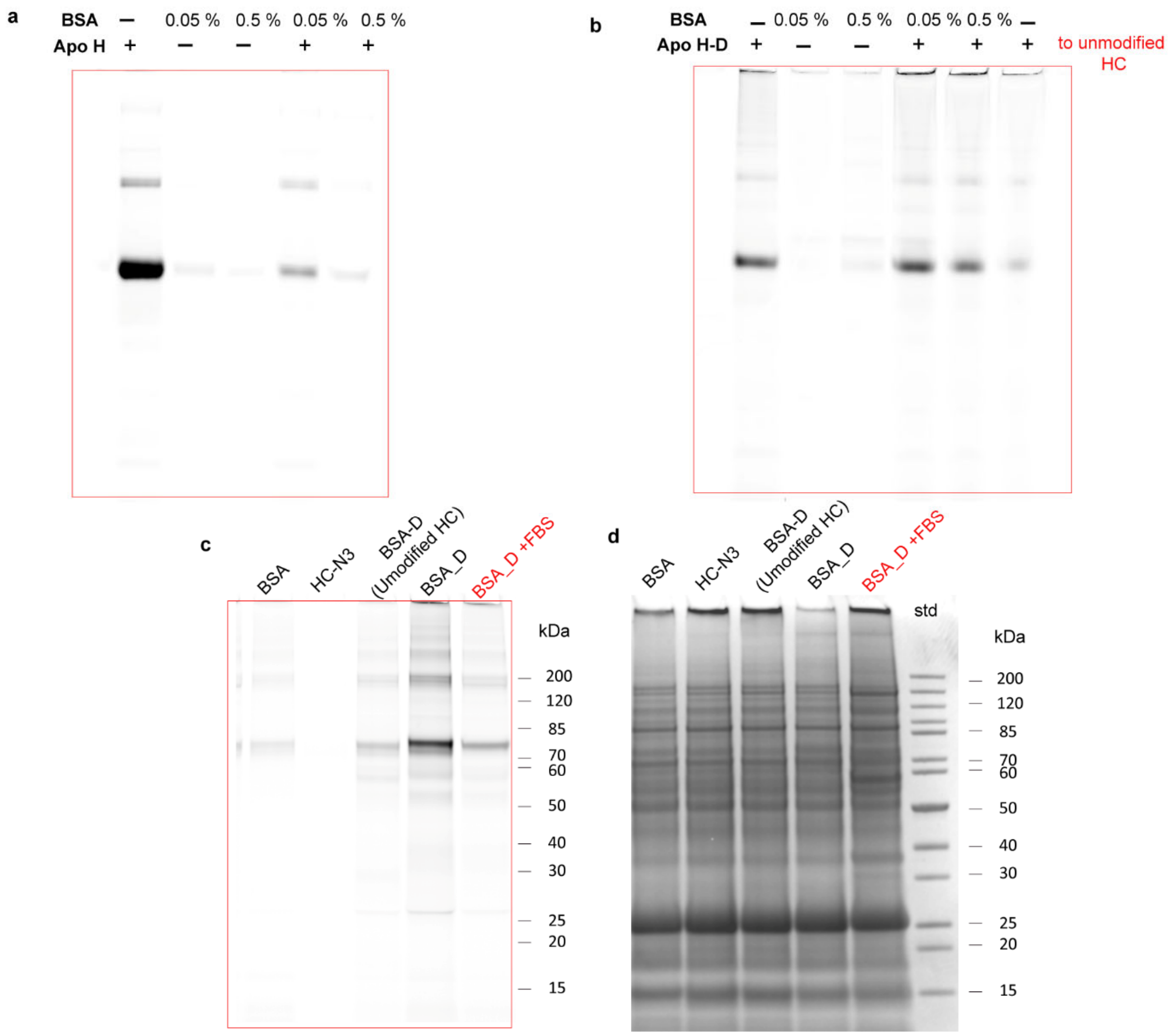

Supplementary Figure 13. The complete SDS-PAGE of competition study shown in Fig. 4. a, Fluorescence image of SDS-PAGE gel of proteins eluted from nanoparticles in the experiment of the addition of fluorescently labeled APO H to SNPs (**a**) and APO H-D to SNPs@HC-N_3 (**b**) in the presence of varying concentrations of BSA (0.05 and 0.5 %). **c,d,** Fluorescence image (**c**) and coomassie stained image (**d**) of the addition of fluorescently labeled BSA to SNPs@HC-N_3 in the presence and absence of FBS proteins. Similar results were obtained in 3 independent experiments. Source data are provided as a Source Data file.

Supplementary Figure 14.

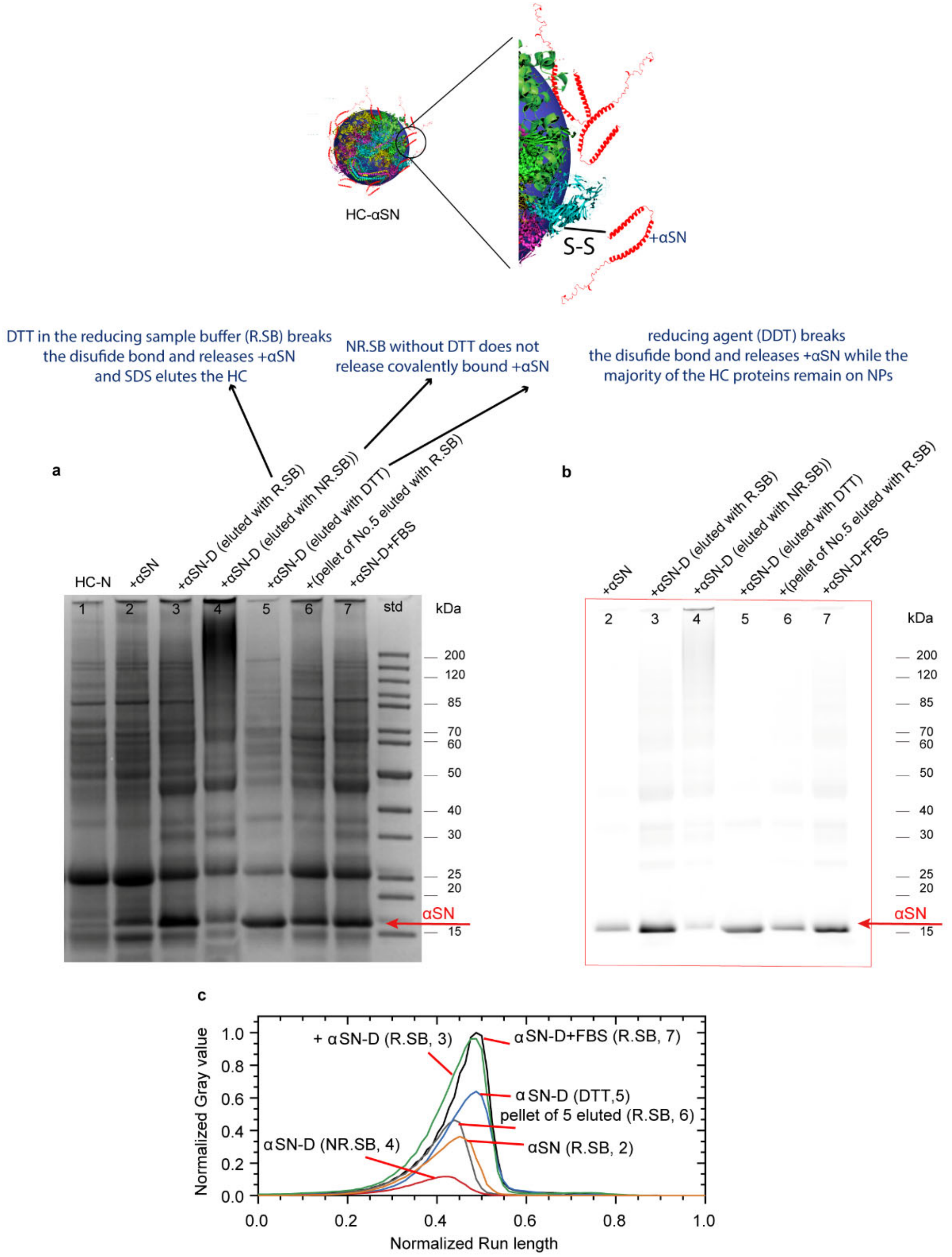

Supplementary Figure 14. Capturing fluorescently labeled α-Synuclein (αSN) as a disease-related protein on HC on SNPs by click chemistry reaction. The corona proteins were eluted with different elution buffers, reducing sample buffer (R.SB, containing SDS, DTT, and glycerol), non-reducing sample buffer (NR.SB, containing SDS and glycerol), and DTT alone as a reducing agent. **a-c,** Coomassie stained (**a**) and fluorescent image (**b**) of the SDS-PAGE gel, and densitometry analysis of the fluorescent image of SDS-PAGE gel of proteins eluted from SNPs. Numbers in the images are as follows: 1) HC-N_3 (eluted with R.SB), 2) αSN added to HC-N_3 on SNPs (eluted with R.SB), 3) αSN-D added to HC-N3 on SNPs (eluted with R.SB), 4) αSN-D added to HC-N_3 on SNPs (eluted with NR.SB), 5) αSN-D added to HC-N_3 on SNPs (eluted with DTT alone), 6) proteins on the pellet of No.5 was eluted with R.SB, 7) αSN-D added to HC-N_3 on SNPs in the presence of FBS (eluted with R.SB). The results confirm that DTT is necessary to reduce the disulfide bridge in the Sulpho-SASD structure to release the majority of αSN. Similar results were obtained in 3 independent experiments. Source data are provided as a Source Data file.

Supplementary Figure 15.

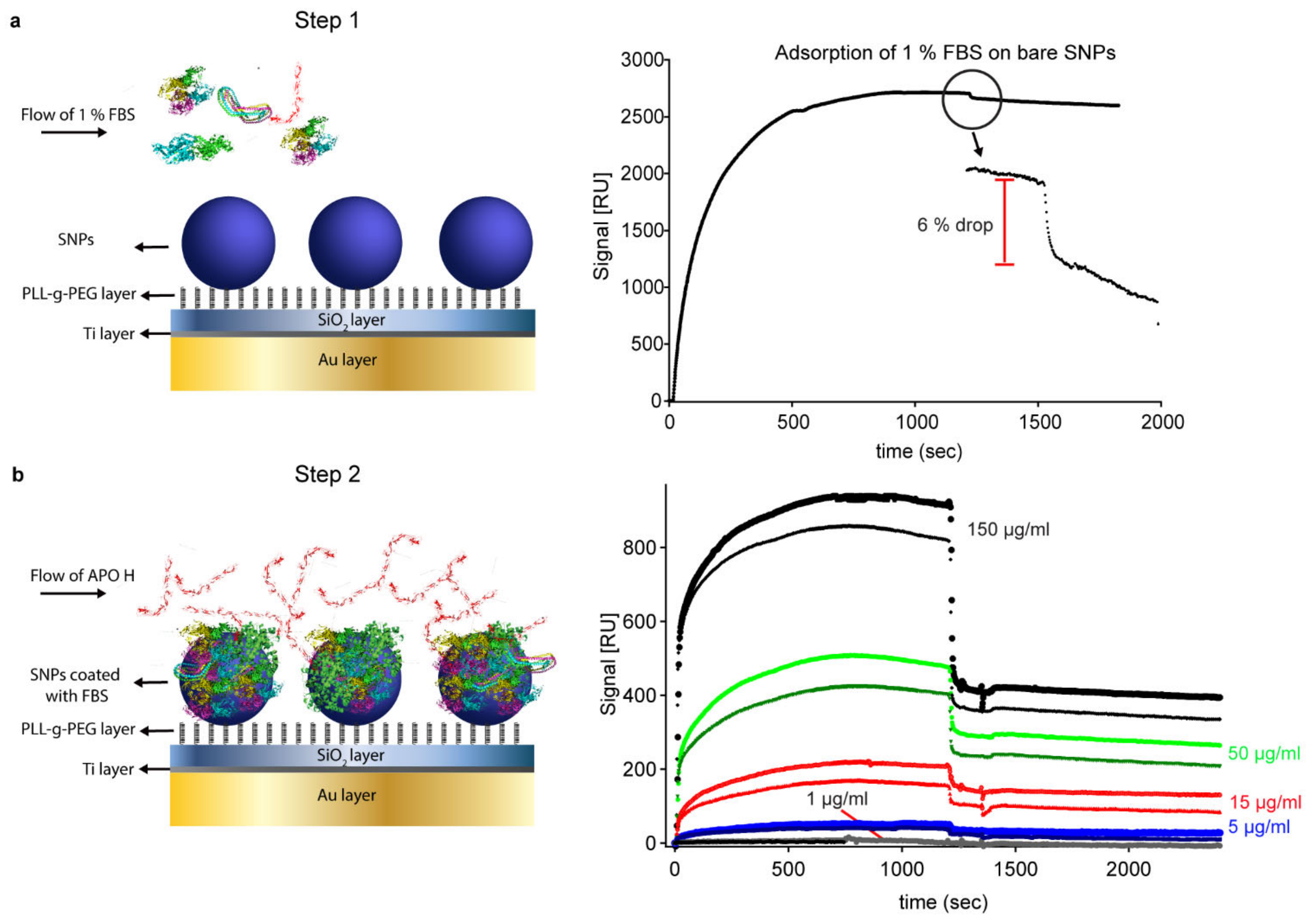

Supplementary Figure 15. SPR measurements. a, SPR measurements on SNPs on PLL-g-PEG with injections of 1 % FBS. The protein corona was formed by injecting 1 % FBS onto the immobilized SNPs. The FBS binds to the NPs, and reaches a stable plateau during this injection signifying equilibrium has been reached. The signal only drops 6% when rinsing commences, indicating that the proteins are tightly adsorbed to the NPs. **b,** Injection of 1, 5, 15, 50 and 150 µg ml^{-1}APO H following the FBS injection. A rapid drop of up to 60 % of the adsorbed APO H was seen in 50 µg ml^{-1}APO H immediately upon rinsing. This indicates that a fraction of the APO H associates with the NPs as part of the soft corona that is instantly removed by rinsing. A fraction of the APO H stays associated with the NPs even after rinsing, which was also expected based on a gel with HC + APO H incubation (Fig.4). Source data are provided as a Source Data file.

Supplementary Figure 16.

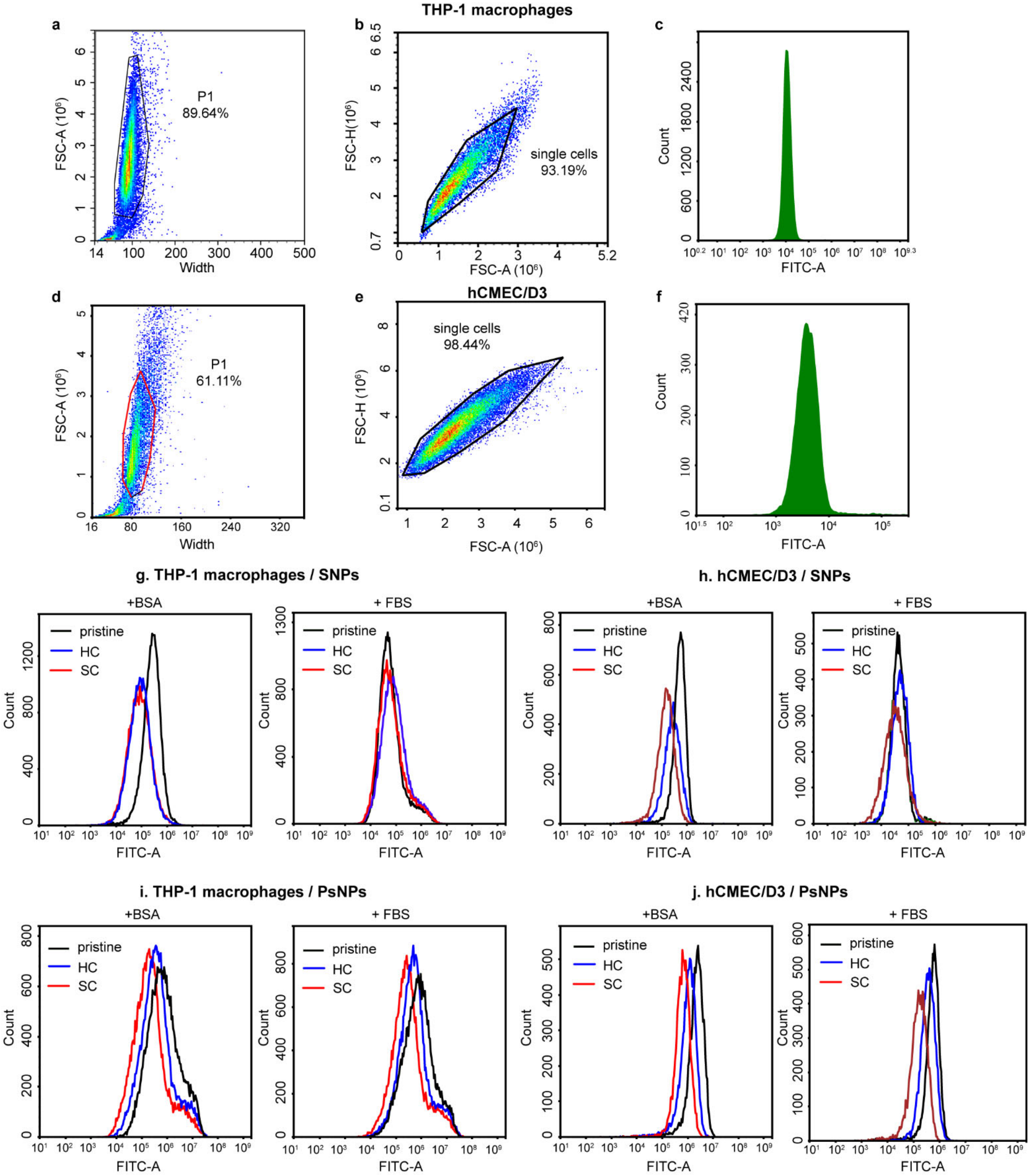

Supplementary Figure 16. Flow cytometry-gating strategy for cell association of nanoparticles. a, THP-1 macrophage cell debris were excluded in a forward scatter/ width dot plot (FSC-A vs width) and the gate was applied to the samples. **b,** A FSC-H vs FSC-A dot plot was used to select THP-1 macrophage single cells. **c**, The median fluorescence intensity of control samples (untreated with nanoparticles) was determined via a histogram. **d-f,** The same strategy as what used for THP-1 macrophage cells was also applied to the hCMEC/D3 cells. **g,h,** The median fluorescence intensity of SNPs associated with THP-1 macrophages (**g**) and hCMEC/D3 cells (**h**) in RPMI media supplemented with BSA or FBS. **i, j**, The median fluorescence intensity of PsNPs associated with THP-1 macrophages (**i**) and hCMEC/D3 cells (**j**) in RPMI media supplemented with BSA or FBS. Source data are provided as a Source Data file.

Supplementary Figure 17.

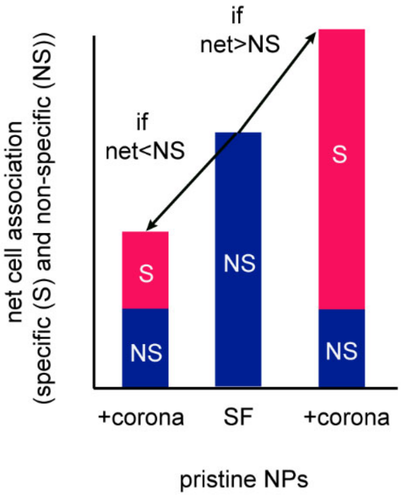

Supplementary Figure 17. net effect of non-specific (NS) interactions and specific interactions (S). The final cell association of the nanoparticle-corona complexes will be determined by the net effect of non-specific (NS) interactions of the particle surface with the cells and specific interactions (S) with the HC, which suggests that the properties of the bare particle surface can still directly influence cell association after a protein corona is formed. Depending on the number of specific interactions made by HC, the final net cell association can be more or less than the non-specific interaction of pristine nanoparticles in the serum-free (SF) medium.

Supplementary Figure 18.

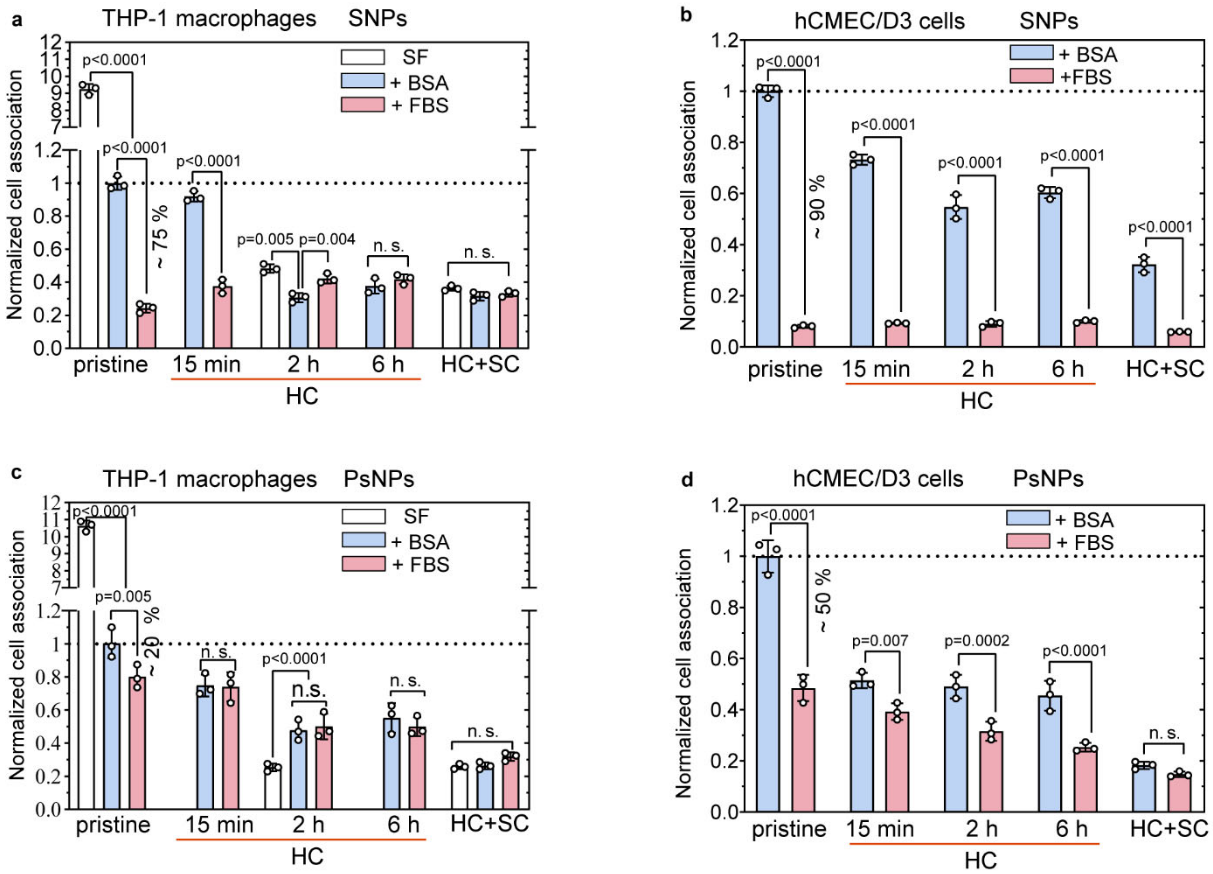

Supplementary Figure 18. Comparison of cell association of SNPs-corona complexes and PsNPs-corona complexes in RPMI supplemented with BSA and FBS, shown in Fig.5. a ,b, Comparison of the cell association of SNPs-corona complexes (**a**) and PsNPs-corona complexes (**b**) in THP-1 macrophages. **c,d,** Comparison of the cell association of SNPs-corona complexes (**c**) and PsNPs-corona complexes (**d**) in hCMEC/D3 cells. The cells were exposed to the pristine NPs, NPs coated with HC formed over different FBS exposure times (15 min, 2 h, and 6 h), and NPs coated with HC+SC for four hours in serum-free RPMI or supplemented with 0.5 % BSA or 10 % FBS. The flow cytometry data were normalised to the pristine nanoparticles values in the RPMI supplemented 0.5 % BSA. Bars show mean ± sd. of three biologically independent experiments (n=3). For the multiple comparison, P value was calculated by one-way ANOVA with Tukey Post hoc test without any adjustment. n.s., not significant (p>0.05). Source data are provided as a Source Data file.

Supplementary Figure 19.

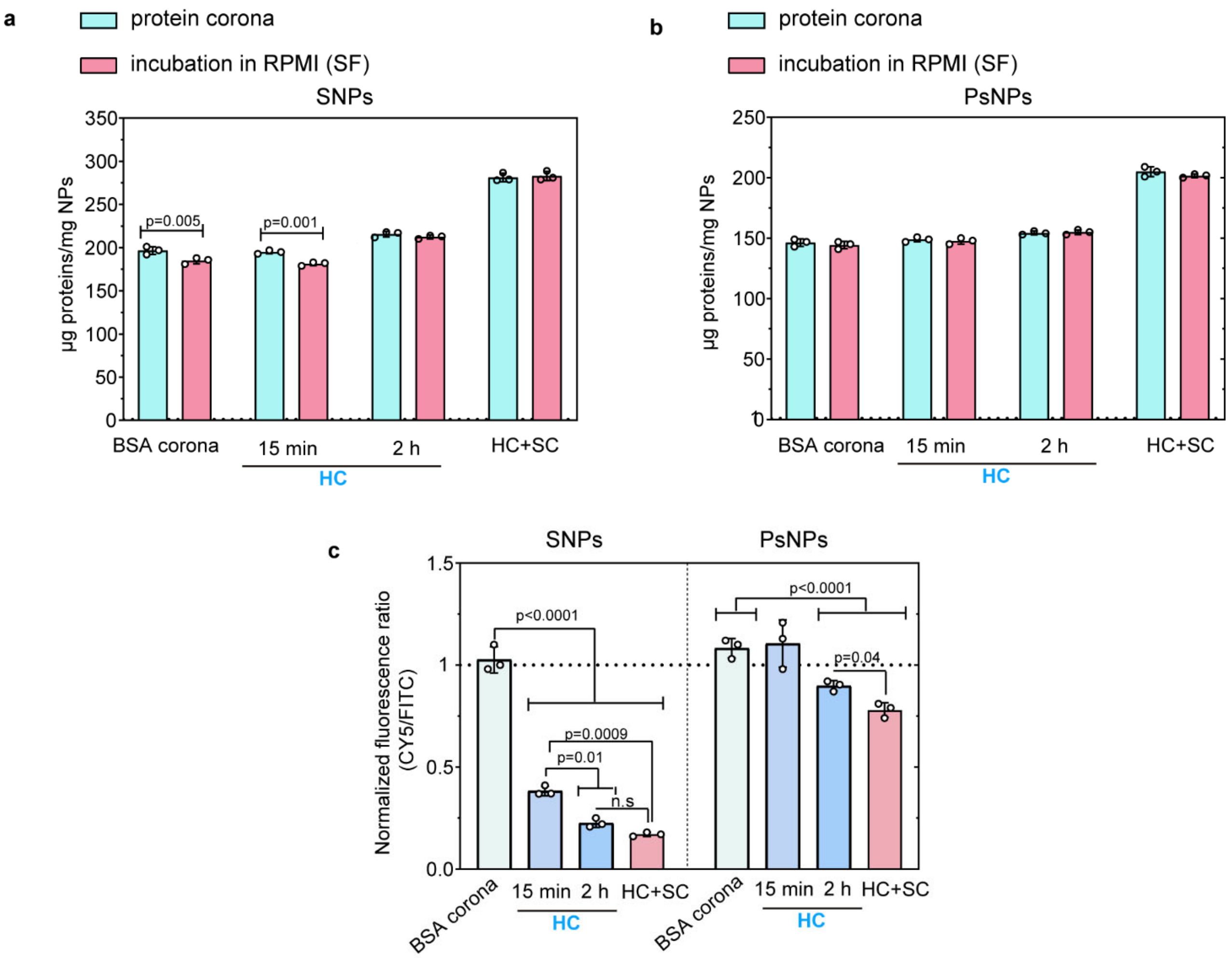

Supplementary Figure 19. Stability of proteins on nanoparticles. a,b, Stability of corona proteins on SNPs (**a**) and PsNPs (**b**) in a serum-free RPMI medium. The nanoparticles with BSA corona, FBS corona (HC), and HC+SC were incubated in RPMI (SF) for 2 h. BSA corona and HC_15 min on SNPs were less stable than HC_2h and HC-SC, while all protein coronae on PsNPs were stable. **c,** Exchange of corona proteins on SNPs and PsNPs with CY5 labeled BSA (BSA-CY5). 5 mg ml^{-1} BSA-CY5 was added to the nanoparticles with different corona (BSA, HC_15 min, HC_2 h, and HC+SC) and incubated for 2 h. The CY5 fluorescence is representative of BSA proteins exchanged with or added to preformed proteins and FITC fluorescence is for nanoparticles. The fluorescence data were normalized to the BSA corona values on SNPs. Bars show mean ± sd. of three biologically independent experiments (n=3). For the multiple comparison, P value was calculated by two-way ANOVA with Tukey Post hoc test without any adjustment. n.s., not significant (p>0.05). Source data are provided as a Source Data file.

Supplementary Table 1. Degree of labelling (DOL) of proteins with DBCO Sulpho-NHS and Sulpho-NHS CY5. The degree of labelling of proteins were calculated by equation 1, using the UV-Vis absorbance of samples at 280 nm, 309 nm, and 646 nm for proteins, DBCO, and CY5, respectively. Data shown correspond to mean ± sd. of three independent experiments (n=3).

	**Condition**		**Degree of labeling (DOL)**	
	**DBCO (mM)**	**Sulpho CY5 (mM)**	**DBCO**	**CY5**
**FBS-D**	0.2	0	4.2±0.3	-
	0.4	0	5.1±0.2	-
	0.8	0	5.6±0.1	-
**FBS-CY5**	0	0.13	-	1.1±0.08
**FBS-D-CY5**	0.4	0.13	4.9±0.1	1.2±0.1

Supplementary Table 2. Characterization of nanoparticle-corona complexes in buffer. The average size of nanoparticle-corona complexes was determined using DLS and the zeta potential measurement data processing was done by using Smoluchowski model. Zeta potential measurement was done in 10 mM sodium phosphate buffer, pH 7.4, containing 10 mM NaCl. Data shown correspond to mean ± sd. of three independent experiments (n=3).

	nanoparticle-corona complexes	zeta potential ± SD (mV)	hydrodynamic diameter ± SD (nm) (PDI)
**SNPs**	pristine	-14±2.1	79±2.8 (0.03)
	HC	-13±1.8	101±5.3 (0.05)
	HC-N_3	-13±1.3	115±10.2 (0.11)
	D Ctrl	-14±2.5	117±7.8 (0.14)
	N_3 Ctrl	-16±1.7	127±6.3 (0.16)
	HC+SC	-17±2.9	148±9.1 (0.18)
**PsNPs**	pristine	-38±2.4	82±3.5 (0.02)
	HC	-33±2.1	109±4.2 (0.04)
	HC-N_3	-36±1.9	119±5.8 (0.09)
	D Ctrl	-30±0.9	123±4.1 (0.10)
	N_3 Ctrl	-29±1.3	129±7.8 (0.09)
	HC+SC	-31±1.8	156±8.3 (0.12)

Supplementary Table 3. Kinetic parameters of binding of APO H to HC proteins on SNPs. Two-dimensional fits were applied to the SPR data to achieve values for K_d and K_{off} for different populations of APO H binding to the HC on SNPs.

Population	% of total signal	K_{off} (s^{-1})	K_d (M)
**1**	88 ± 4.4	$4.8\times10^{-2} \pm 8.0\times10^{-3}$	$1.7 \times10^{-5} \pm 3.6\times10^{-6}$
**2**	9.54 ±3.46	$8.1\times10^{-5} \pm 4.9\times10^{-6}$	$7.3\times10^{-8} \pm 6.6\times10^{-9}$

Supplementary Table 4. Size distribution of nanoparticle-corona complexes in cell culture medium (RPMI+10 % FBS). The average size of nanoparticle-corona complexes was determined using DLS Data in the cell culture medium (RPMI+10 % FBS). Data shown correspond to mean ± sd. of three independent experiments (n=3).

	nanoparticle-corona complexes	DLS hydrodynamic diameter ± SD (nm) (PDI)
**SNPs**	pristine	112 ±6.3 (0.10)
	HC	138±7.7 (0.11)
	HC+SC	161±9.4 (0.21)
**PsNPs**	pristine	145±7.4 (0.09)
	HC	165±8.7 (0.1)
	HC+SC	203±10.1 (0.33)
