# Supplementary material for: Mapping and identification of soft corona proteins at nanoparticles and their impact on cellular association
Source: Nat Commun. 2020 Sep 10;11:4535. doi: 10.1038/s41467-020-18237-7 (PMC7484794; doi:10.1038/s41467-020-18237-7)
Supplement: Supplementary file 3 — Description of Additional Supplementary Files [file 41467_2020_18237_MOESM3_ESM.docx]

**Description of Additional Supplementary Files**

File Name: Supplementary Data 1

Description: Total corona proteins detected in all samples including their accession number and protein parameters
