# Supplementary material for: Mapping and identification of soft corona proteins at nanoparticles and their impact on cellular association
Source: Nat Commun. 2020 Sep 10;11:4535. doi: 10.1038/s41467-020-18237-7 (PMC7484794; doi:10.1038/s41467-020-18237-7)
Supplement: Supplementary file 5 — Reporting Summary [file 41467_2020_18237_MOESM5_ESM.pdf]

nature research

Corresponding author(s): Duncan Sutherland

Last updated by author(s): Aug 6, 2020

Reporting Summary

Nature Research wishes to improve the reproducibility of the work that we publish. This form provides structure for consistency and transparency in reporting. For further information on Nature Research policies, see Authors & Referees and the Editorial Policy Checklist .

Statistics

For all statistical analyses, confirm that the following items are present in the figure legend, table legend, main text, or Methods section.

n/a	Confirmed	
☐	☒	The exact sample size (*n*) for each experimental group/condition, given as a discrete number and unit of measurement
☐	☒	A statement on whether measurements were taken from distinct samples or whether the same sample was measured repeatedly
☐	☒	The statistical test(s) used AND whether they are one- or two-sided<br>*Only common tests should be described solely by name; describe more complex techniques in the Methods section.*
☒	☐	A description of all covariates tested
☒	☐	A description of any assumptions or corrections, such as tests of normality and adjustment for multiple comparisons
☐	☒	A full description of the statistical parameters including central tendency (e.g. means) or other basic estimates (e.g. regression coefficient) AND variation (e.g. standard deviation) or associated estimates of uncertainty (e.g. confidence intervals)
☐	☒	For null hypothesis testing, the test statistic (e.g. *F*, *t*, *r*) with confidence intervals, effect sizes, degrees of freedom and *P* value noted<br>*Give P values as exact values whenever suitable.*
☒	☐	For Bayesian analysis, information on the choice of priors and Markov chain Monte Carlo settings
☐	☒	For hierarchical and complex designs, identification of the appropriate level for tests and full reporting of outcomes
☒	☐	Estimates of effect sizes (e.g. Cohen's *d*, Pearson's *r*), indicating how they were calculated

Our web collection on statistics for biologists contains articles on many of the points above.

Software and code

Policy information about availability of computer code

Data collection	Flow cytometry=NovoExpress software (v.1.4.1)<br>Confocal images= Zen (ZEISS) (v.2.6)
Data analysis	GraphPad Software (v.8.2.1, La Jolla California USA), R environment (v. 3.5.1) and R packages; FactoMineR (v. 1.41), factoextra (v. 1.0.5), gplots (v. 3.0.1.1) and dendextend (v. 1.9.0), Image J (1.51), Microsoft Excel for Windows (2016), Mascot Search Engine (Matrix Science), Biorad gel documentation, MATLAB 2012a platform (Mathworks) using the fitting tool EVILFIT version 3 software , Proteome Discoverer (Thermo Scientific, v.2.4), ProtParam (SIB ExPASY Bioinformatic Resources Portal)

For manuscripts utilizing custom algorithms or software that are central to the research but not yet described in published literature, software must be made available to editors/reviewers. We strongly encourage code deposition in a community repository (e.g. GitHub). See the Nature Research guidelines for submitting code & software for further information.

Data

Policy information about availability of data

All manuscripts must include a data availability statement. This statement should provide the following information, where applicable:

- Accession codes, unique identifiers, or web links for publicly available datasets
- A list of figures that have associated raw data
- A description of any restrictions on data availability

All relevant data that support the plots within this paper and other findings of this study are available with the article and supplementary files or in the Source data file. The source data underlying Figs. 1b-h, 2, 3, 4a-f, 4h,i, 5, 6 as well as Supplementary Figs. 2, 3, 4, 5, 6, 7d, 9, 10, 11, 13, 14, 15, 16a-d, 18, and 19 are provided as a Source Data file. Extra data are available from the corresponding authors upon reasonable request. Bovine proteome and Uniprot Fasta is available on https://www.uniprot.org/. The mass spectrometry proteomics data have been deposited to the ProteomeXchange Consortium via the PRIDE partner repository with the dataset identifier PXD020768. The PDB format for the structure of proteins are available on Protein Data Bank (https://www.rcsb.org). The protein parameters (molecular weight, isoelectric point, GRAVY score, and instability index) were obtained from https://www.expasy.org.

Field-specific reporting

Please select the one below that is the best fit for your research. If you are not sure, read the appropriate sections before making your selection.

☒ Life sciences ☐ Behavioural & social sciences ☐ Ecological, evolutionary & environmental sciences

For a reference copy of the document with all sections, see nature.com/documents/nr-reporting-summary-flat.pdf

Life sciences study design

All studies must disclose on these points even when the disclosure is negative.

Sample size	No sample size calculations were performed for this study as we did not perform any in vivo or patient analysis. No group comparisons were made in order to determine a specified effect. Sample size for in vitro cell cultures and other experiments are indicated in each figure legend. The sample size were chosen so we had enough data to do statistical analysis.
Data exclusions	No data were excluded from analysis; all analyses were performed as described in the Materials and Methods
Replication	All cell data and other findings were obtained by three technical replicates and for most analysis assays were repeated two or three times batches. All experiments findings were reliably reproducible.
Randomization	n/a. This study was not concerned with individuals but with populations of cell lines; however, the differentiated THP-1 cells and hCMEC/D3 cells were randomly chosen from each batch of cultured cells for further analysis.
Blinding	n/a. The investigators were not blind to group allocation during data collection as the data collection and the subsequent analysis were not sensitive to potential bias.

Reporting for specific materials, systems and methods

We require information from authors about some types of materials, experimental systems and methods used in many studies. Here, indicate whether each material, system or method listed is relevant to your study. If you are not sure if a list item applies to your research, read the appropriate section before selecting a response.

Materials & experimental systems

n/a	Involved in the study	
☒	☐	Antibodies
☐	☒	Eukaryotic cell lines
☒	☐	Palaeontology
☒	☐	Animals and other organisms
☒	☐	Human research participants
☒	☐	Clinical data

Methods

n/a	Involved in the study	
☒	☐	ChIP-seq
☐	☒	Flow cytometry
☒	☐	MRI-based neuroimaging

Eukaryotic cell lines

Policy information about cell lines

Cell line source(s)	THP-1 monocyte cells (a human acute monocyte leukemia cell line) was obtained from the German Collection of Microorganisms and Cell Cultures (DSMZ, ACC 16).hCMEC/D3 cells were obtained from sigmaaldrich (SCC066).
Authentication	The cell lines were not authenticated.
Mycoplasma contamination	Negative for Mycoplasma
Commonly misidentified lines (See ICLAC register)	No misidentified cell lines were used.

Flow Cytometry

Plots

Confirm that:

- [x] The axis labels state the marker and fluorochrome used (e.g. CD4-FITC).
- [x] The axis scales are clearly visible. Include numbers along axes only for bottom left plot of group (a 'group' is an analysis of identical markers).
- [x] All plots are contour plots with outliers or pseudocolor plots.
- [x] A numerical value for number of cells or percentage (with statistics) is provided.

Methodology

Sample preparation	The detached cells were washed three times with PBS to remove free nanoparticles. The cells were then fixed by 4 % paraformaldehyde for 15 min. The cells were washed three times with PBS and resuspended in 200 ul PBS. In the flowcytometry analysis, at least 10000 cells were counted. The fluorescent dara are represented as median and calculated as the ratio of median fluorescent intensity of the samples and the pristine nanoparticles in BSA containing medium.
Instrument	NovoCyte flow cytometer
Software	NovoExpres software (v.1.4.1)
Cell population abundance	cells were not sorted
Gating strategy	Cell debris and doublets were first excluded in a forward scatter/ width density plot (FSC-A vs width) and then in a FSC-H vs FSC-A density plot to select a population of single cells.

- [x] Tick this box to confirm that a figure exemplifying the gating strategy is provided in the Supplementary Information.
